# CRISP-ID: decoding CRISPR mediated indels by Sanger sequencing

**DOI:** 10.1038/srep28973

**Published:** 2016-07-01

**Authors:** Jonas Dehairs, Ali Talebi, Yacine Cherifi, Johannes V. Swinnen

**Affiliations:** 1 Laboratory of Lipid Metabolism and Cancer, Department of Oncology, KU Leuven, 3000 Leuven, Belgium; 2 genOway, Lyon F69007, France

## Abstract

The advent of next generation gene editing technologies has revolutionized the fields of genome engineering in allowing the generation of gene knockout models and functional gene analysis. However, the screening of resultant clones remains challenging due to the simultaneous presence of different indels. Here, we present CRISP-ID, a web application which uses a unique algorithm for genotyping up to three alleles from a single Sanger sequencing trace, providing a robust and readily accessible platform to directly identify indels and significantly speed up the characterization of clones.

Whilst the next generation gene editing tools, zinc finger nucleases and TALENs have been widely available^1^,^2^, the advent of the CRISPR-Cas9 system (CRISPR) augmented the accessibility of precise gene editing, leading to its ubiquitous adoption. CRISPR allows the rapid generation of gene knockouts or knock-ins in *in vitro* and in *in vivo* models, and finds a wide range of applications beyond gene editing^3^,^4^,^5^.

The CRISPR mode of action has been previously described in great detail. Briefly, CRISPR is a bipartite system comprised of an endonuclease domain entailing the Cas9 protein and a guide RNA (gRNA), which binds Cas9. The gRNA variable domain can be modified to target virtually any gene of interest, thereby localizing the system to a specific region of the genome. Depending on the nature of the Cas9 protein, this results in a DNA double strand break or a nick, leading to nucleotide insertions or deletions (indels) due to errors in the cell’s endogenous DNA repair mechanisms. Alternatively, if an oligo-nucleotide with a high degree of homology surrounding the strand break or nicks is introduced, the endogenous homology directed repair mechanism can use the oligo as a repair template, thereby allowing precise gene insertions or modifications^6^.

In a diploid cell, next generation nuclease-mediated gene-silencing commonly results in either one or two indels. Since the indels typically introduced by the repair mechanisms are largely random, they are unlikely to be identical. Even in diploid cells, three different indels are often observed. These three indels can arise from colony formation that started from two cells as opposed to one, or more likely as a result of residual nuclease activity in a daughter cell resulting in an additional indel as this phenomenon is observed even under strict single cell sorting conditions.

In order to identify the exact sequence of the resulting alleles in selected clones, most laboratories use Sanger sequencing. Typically, the targeted exon is PCR amplified and cloned into a vector for bacterial single colony sequencing. Although this is considered the gold standard, this method can be costly, time consuming and laborious, even for a limited number of clones. Alternatively, the PCR product can be sequenced directly by Sanger sequencing but this results in a convoluted spectrum with overlapping peaks that is difficult to delineate with current methodologies.

Whilst several tools have been developed to de-convolute spectra with overlapping peaks arising from heterozygous indels (Indelligent, CHILD, Mixed Sequence Reader, etc.)^7^,^8^,^9^, these tools are either unable to directly read trace files, are no longer available, are not available as a web application and were not designed to interpret overlapping spectra arising from more than two different alleles. Furthermore, these tools predate the advent of CRISPR technologies and are unable to correctly identify CRISPR mediated indels.

Here we present CRISP-ID, a web-based application for identifying indels through direct Sanger sequencing of PCR products. Although here we focus on CRISPR-induced indels (due to its ubiquitous adoption), this tool is also applicable to zinc finger nucleases, TALENs and the analysis of frame-shift mutations in cancer or rare genetic disorders. CRISP-ID directly reads sequencing trace files (ABI and SCF files) and is the first application with the ability to de-convolute the overlapping spectra from three different alleles, providing a robust and easy to use clone identification tool using direct standard Sanger sequencing of PCR products from cell line clones or patient material, without bacterial sub-cloning.

## Results

**Figure 1:**
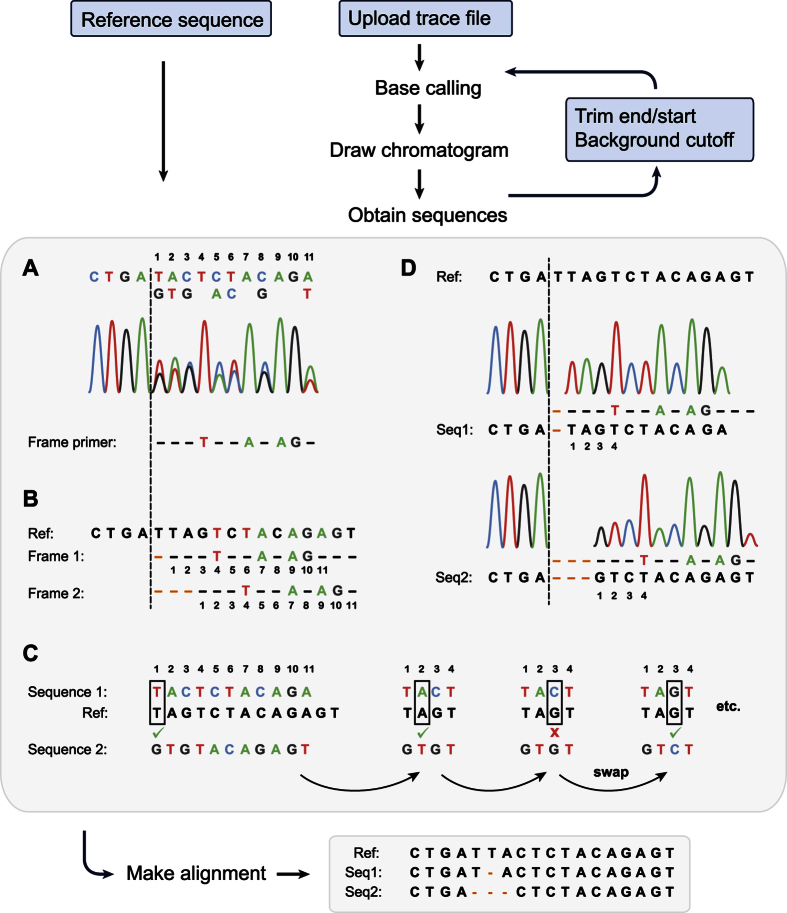
Input, processing and output of the CRISP-ID application. After uploading a trace file, CRISP-ID draws a chromatogram that displays the sequence peaks and base calls. The user can tweak the base calling by trimming the start and the end of the chromatogram and by adjusting the background cut-off. To obtain the sequences, a “frame primer” is constructed, containing only the homozygous base calls after the start of the spectral shift (**A**). This frame primer runs over the entire length of the overlapping spectrum. Provided that the frame primer is sufficiently long, it will align with the reference sequence once for each sequence that is present in the mix (**B**). If no alignments can be found initially, the primer is iteratively trimmed until either alignments are found, or a minimal size of 10 homozygous base calls is reached. Trimming the 3′ end of the frame primer might be necessary due to poor quality base calls near the end of the sequence run, trimming the 5′ end is necessary in case of insertions, and is set to 10 bases by default (not shown in figure). Initially, a “first guess” of the sequences is constructed based on peak height, with the first sequence containing the highest peaks (**C**). The first sequence is then compared to the reference sequence according to the first frame found in step B. In case of a base mismatch, the base is swapped with the second sequence. During this process of matching the first sequence to the reference sequence, the swapping inherently results in simultaneously solving the second sequence (**D**). Finally, the user is presented with a multiple alignment of the de-convoluted sequences and the reference sequence, revealing the size and locus of the indels.

To identify indels directly from Sanger sequencing traces of PCR products without sub-cloning, we developed CRISP-ID. CRISP-ID uses the BioJava API^10^ to import trace files and uses a unique, newly developed algorithm to de-convolute up to three overlapping spectra (Fig. 1). Homozygous base calls following the spectral shift are used to align the overlapping spectra with a reference sequence. Typically, fewer than 100 peaks following the frame shift provide sufficient information to uniquely align each overlapping spectrum to the reference sequence and reveal its sequence. The user is given the option of excluding base-calls from the start and end of the Sanger sequence reads as confidence in base calls in this region can be low. Finally, an alignment of the resolved sequences with the reference sequence is presented to reveal the exact size and the location of the indels.

In order to demonstrate the applicability of CRISP-ID, we used CRISPR-Cas9 to knockout genes in both *in vitro* and in an *in vivo* model. The *ELOVL6*, *MBTPS1* and *SREBF1* genes were knocked out in a diploid human cell line, *Elovl6* in a diploid mouse derived cell line and *Fxr1* in an *in vivo* mouse model. The targeted exons were amplified using a high fidelity proofreading DNA polymerase. The PCR products were sequenced directly by Sanger sequencing and as single colonies following bacterial cloning. A total of 3–6 randomly selected clones per gene (depending on clone availability) were analyzed for each cell line. Fourteen clones contained two alleles and eight had three alleles for the genes of interest. The sequence identity of the first 200 bases following the spectral shift (or until the end of the sequence run, if fewer than 200 bases were covered) was on average 99.9% identical to the single colony method. The small uncertainty in the base calling is likely due to the presence of poor quality peaks from the Sanger sequencing data, or due to random insertions or substitutions in the same locations in different alleles which couldn’t be traced back to the correct allele. These rare mistakes (<0.10%) were found to have no effect on the determination of the indel size and locus, which matched perfectly to the single colony method (Table 1).

## Discussion

Next generation gene editing tools provide powerful and widely adopted techniques for the rapid generation of knockout and knock-in models, which is readily accessible to any lab. There is however no correspondingly facile tool for resultant clone characterization. Next generation sequencing (NGS) can be applied in some cases. The most commonly used NGS platforms are however hampered by short read lengths and by multiplexing. Multiplexing requires the addition of uniquely tagged primers for each clone. With single molecule real time sequencing, read lengths are no longer an issue, but this technique still requires the generation of unique tags. Furthermore, NGS remains inaccessible and impracticable based on the throughput of most laboratories, creating a discrepancy between the ready accessibility of next generation gene editing tools, but the paucity of accessible tools in clone characterization. There remains a clear need for a widely applicable and accessible method for clone characterization.

Sanger sequencing is commonly used to characterize indels, however de-convolution of spectra from mixed alleles remains challenging and can be overcome by cloning the resultant alleles in bacteria, however this is laborious and costly.

CRISP-ID provides a facile and commonly available method for the unequivocal characterization of the indels from resultant clones by allowing the exact determination of resultant indels in diploid or triploid cells directly from Sanger sequencing of PCR products. CRISPR-ID cannot be used to identify more than three indels from one sequencing run. This is both due to the low probability of homozygous base-calls and due to the technical reality that higher trace numbers degrade the spectrum quality. CRISP-ID uses an intuitive graphical interface making it widely accessible. The software has been validated for CRISPR-Cas9-mediated knockouts of genes in a diploid human cell line, a diploid mouse cell line and an *in vivo* mouse model, and has been found to perfectly discriminate up to three alleles without the need of sub-cloning of PCR products. This results in a substantial reduction in time and costs. The software is freely available at: http://crispid.gbiomed.kuleuven.be.

## Material and Methods

### Cell culture

A diploid human melanoma cell line (451LU) and a mouse melanoma derived cell line (FLCM) were cultured in DMEM (Sigma - D6546) supplemented with 10% FBS (Life Technologies) and 4 mM glutamine (Life technologies - 25030–081). Cells were periodically checked for mycoplasma contamination and were mycoplasma free.

### Transfections

Plasmid constructs for CRISPR-Cas9 coupled to GFP with a guide-RNA targeting mouse *Elovl6* exon 5 and human *SREBF1* exon 1 were purchased (Sigma). The following constructs were designed using E-CRISP: human *ELOVL6* exon 2: GTGCCGACCACCGAATATAAAGG, human *MBTPS1* exon 1: GTGGGAACAGCCAGGGCATG. The annealed oligos were ligated into pSpCas9(BB)-2A-GFP (PX458) as previously described^11^. The cell lines were transfected with the plasmid (Neon Transfection System, Life Technologies). 72 hours post-transfection, the top 10% of GFP expressers were sorted by FACS (BD bioscience, ARIA III) into single wells for colony formation. Dead cells were excluded from the sort by the membrane exclusion dye Sytox Blue (Life Technologies - S34857).

### Fxr1 KO mouse

The *Fxr1* gene was targeted at exon 15, CRISPR: CAGGCAGAAGATAGACAGCC. Cas9 mRNA was generated by *in vitro* transcription from the T7 promoter using the HiScribe T7 ARCA mRNA kit from NEB (#E2060S) whereas the guide-RNA was transcribed using the MEGAshortscript T7 transcription kit from Thermo Fisher (#AM1354). The Cas9 mRNA and the guide-RNA were co-injected into fertilized oocytes from C57BL/6N mice. Oocytes were transferred to the oviducts of pseudo-pregnant mice. The resulting mice were crossed with wild-type C57BL/6N mice and material was obtained from their offspring. Mice were obtained from Charles River (Charles River Laboratories, France) and the experiments were approved by the local ethical committee for animal experiments (Charles River ethical committee for animal experiments) in accordance with EU/2010/63 and AAALAC guidelines.

### PCR and cloning

The targeted exons were PCR amplified using Platinum Pfx DNA Polymerase (Life Technologies) with the following primers: mouse *Elovl6* exon 6: Fw 5′-GGCCATCCACCAAGTATGTGAG-3′, Rv 5′-CCGTGCTTTGAGATAAGAGTTGC-3′, human *ELOVL6* exon 2: Fw 5′- GCCGTGTAGACTAGACTCCC-3′, Rv 5′-CAAATGGTGGCAGTGAAGGC-3′, human *SREBF1* exon 1: Fw 5′-CGCGAGGCTGGATAAAATGAAT-3′, Rv 5′-GAGACAAAGGCCAGGGAGAC-3′, human *MBTPS1* exon 1: Fw 5′-AACCCCATTGGACGTTGGTT-3′ Rv 5′-GAAAAGAGGAACATGTTATTCAGCA-3′, mouse *Fxr1* exon 15: Fw 5′- AATGAGAATGGGCTAGGTATGTAAGCACTTAGG-3′, Rv 5′- TCAACCTCAACACAATTCACACCATAGTCC-3′. The forward primers were also used for Sanger sequencing (LGC Genomics, Germany). The PCR products were cloned into pJET1.2/blunt using the CloneJET PCR cloning system (Life Technologies) according to manufacturer’s instructions.

## Additional Information

**How to cite this article**: Dehairs, J. *et al.* CRISP-ID: decoding CRISPR mediated indels by Sanger sequencing. *Sci. Rep.* **6**, 28973; doi: 10.1038/srep28973 (2016).

## Figures and Tables

**Table 1:** Validation of CRISP-ID compared to single colony cloning.

Clone	Indel location/size CRISP-ID	Indel location/size single colony	base identity (%)
*Elovl6*_#1	83_163 del	identical	100
	157 del	identical	100
	157_158 Ins 1	identical	100
*Elovl6*_#2	157_158 del	identical	100
	157 del	identical	100
	157_158 Ins 1	identical	100
*Elovl6*_#3	157_158 Ins 1	identical	100
	157_162 del	identical	100
*Elovl6*_#4	157_160 del	identical	100
	157_165 del	identical	100
*Elovl6*_#5	156_158 Ins 1	identical	100
	157_160 del	identical	99.5
*Elovl6*_#6	152_161 del	identical	100
	157 del	identical	99.5
	157_158 Ins 1	identical	99
*ELOVL6*_#1	40_42 del	identical	100
	40_41 del	identical	100
*ELOVL6*_#2	41_42 del	identical	100
	40_42 del	identical	100
*ELOVL6*_#3	42_45 del	identical	100
	42_43 del	identical	100
	42 del	identical	100
*ELOVL6*_#4	40_45 del	identical	100
	43_44 del	identical	100
*ELOVL6*_#5	42_43 del	identical	100
	42 del	identical	100
	42 Ins 1	identical	100
*ELOVL6*_#6	41_42 del	identical	100
	41 del	identical	100
*SREBF1*_#1	17_20 del	identical	100
	13 del	identical	100
	19_20 Ins 1	identical	99.3
*SREBF1*_#2	20_28 del	identical	100
	19_20 Ins 1	identical	100
*SREBF1*_#3	20_28 del	identical	100
	19_20 Ins 1	identical	100
*SREBF1*_#4	−29_18 del	identical	100
	17 del	identical	100
*SREBF1*_#5	22_29 del	identical	100
	28_29 Ins 1	identical	98.5
	28_29 Ins 6	identical	99.3
*MBTPS1*_#1	104_151 del	identical	100
	105_151 del	identical	100
*MBTPS1*_#2	104_151 del	identical	100
	104_150 del	identical	100
	104_149 del	identical	100
*MBTPS1*_#3	105_154 del	identical	100
	105_151 del	identical	100
*Fxr1*_#1	wild type	identical	99.5
	66_84 del	identical	100
*Fxr1*_#2	wild type	identical	100
	75_76 Ins 1	identical	100

22 single cell-derived clones with a CRISPR-Cas9 mediated KO were analyzed by Sanger sequencing and CRISP-ID, or by the single colony method followed by Sanger Sequencing.
